# Enhancing drought tolerance in cauliflower (*Brassica oleracea* var. *botrytis*) by targeting LFY transcription factor modulation via the ethylene precursor, ACCA: an innovative computational approach

**DOI:** 10.3389/fpls.2024.1255979

**Published:** 2024-02-28

**Authors:** Sandip Debnath, Abdallah M. Elgorban, Ali H. Bahkali, Rajalakshmanan Eswaramoorthy, Meenakshi Verma, Asad Syed, Vetriselvan Subramaniyan, Chinnaperumal Kamaraj, Ling Shing Wong, Vinoth Kumarasamy

**Affiliations:** ^1^ Department of Genetics and Plant Breeding, Institute of Agriculture, Visva-Bharati University, Sriniketan, West Bengal, India; ^2^ Department of Botany and Microbiology, College of Science, King Saud University, Riyadh, Saudi Arabia; ^3^ Department of Biochemistry, Centre of Molecular Medicine and Diagnostics (COMMAND), Saveetha Dental College and Hospitals, Saveetha Institute of Medical and Technical Sciences (SIMATS), Chennai, India; ^4^ University Centre for Research and Development, Department of Chemistry, Chandigarh University, Gharuan, India; ^5^ Jeffrey Cheah School of Medicine and Health Sciences, Monash University, Malaysia, Bandar Sunway, Selangor Darul Ehsan, Malaysia; ^6^ Interdisciplinary Institute of Indian System of Medicine (IIISM), Directorate of Research, SRM Institute of Science and Technology (SRMIST), Chennai, Tamil Nadu, India; ^7^ Faculty of Health and Life Sciences, INTI International University, Nilai, Negeri Sembilan, Malaysia; ^8^ Department of Parasitology and Medical Entomology, Faculty of Medicine, Universiti Kebangsaan Malaysia, Kuala Lumpur, Malaysia

**Keywords:** cauliflower, drought tolerance, yield, ethylene precursor compound, ACCA, agricultural productivity

## Abstract

**Background:**

*Brassica oleracea* var. *botrytis* is an annual or biennial herbaceous vegetable plant in the *Brassicaceae* family notable for its edible blossom head. A lot of effort has gone into finding defense-associated proteins in order to better understand how cauliflower and pathogens interact. Endophytes are organisms that live within the host plant and reproduce. Endophytes are bacteria and fungi that reside in plant tissues and can either help or harm the plant. Several species have aided molecular biologists and plant biotechnologists in various ways. Water is essential for a healthy cauliflower bloom. When the weather is hot, this plant dries up, and nitrogen scarcity can be detrimental to cauliflower growth.

**Objective:**

The study sought to discern plant growth promoting (PGP) compounds that can amplify drought resilience and boost productivity in cauliflower.

**Methods:**

Investigations were centered on endophytes, microorganisms existing within plant tissues. The dual role of beneficial and detrimental *Agrobacterium* was scrutinized, particularly emphasizing the ethylene precursor compound, 1-amino-cyclopropane-1-carboxylic acid (ACCA).

**Results:**

ACCA possessed salient PGP traits, particularly demonstrating a pronounced enhancement of drought resistance in cauliflower plants. Specifically, during the pivotal marketable curd maturity phase, which necessitates defense against various threats, ACCA showcased a binding energy of −8.74 kcal/mol.

**Conclusion:**

ACCA holds a significant promise in agricultural productivity, with its potential to boost drought resistance and cauliflower yield. This could be particularly impactful for regions grappling with high temperatures and possible nitrogen shortages. Future research should explore ACCA’s performance under diverse environmental settings and its applicability in other crops.

## Graphical Abstract

(made in Biorender).
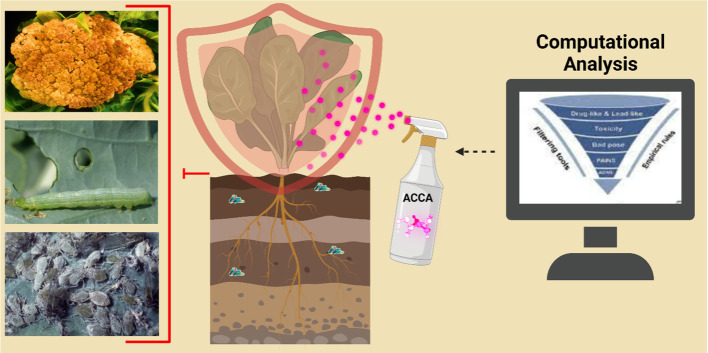

## Introduction

*Brassica oleracea* var. *botrytis*, a member of the *Brassicaceae* family, is an extensively grown herbaceous plant that is renowned for its consumable head consisting of several underdeveloped flowers. Interestingly, these blossoms mostly exhibit female reproductive structures, frequently devoid of male equivalents. The botanical structure has superficial root systems, robust stems, and vividly pigmented light green foliage extending from the stem’s apex. The plant in question normally attains a height of 3.3–4.9 ft. It is widely grown yearly and typically harvested within 60–100 days after being planted. The exact origins of this plant species are elusive, although ancestral conjectures suggest that wild cabbage from ancient Asia Minor may have had a significant role (CABI Crop Protection Compendium, 2008).

From a nutritional standpoint, cauliflower may be considered a highly beneficial food source. The substance contains a significant amount of antioxidants, including glucosinolates, and a variety of vitamins, phenolic compounds, and carotenoids, which are believed to contribute to its beneficial effects on health (Picchi et al., 2020; Lukhovitskaya and Ryabova, 2019). This particular plant is susceptible to several forms of assaults during its life cycle, including pest infestations, microbiological infections, and, notably, the cauliflower mosaic virus. During infections, viruses exploit the cellular machinery of the host cell, with the cauliflower mosaic virus’ translation trans-activator/viroplasmin protein serving as a versatile component that may have the ability to block nonsense-mediated mRNA degradation (Lukhovitskaya and Ryabova, 2019).

For a healthy grown cauliflower flower, water plays a most important role; often due to hot weather, this plant gets dried up, and often, nitrogen scarcity plays a destructive role in growing cauliflowers. LFY encodes a plant-specific transcription factor (Hamès et al., 2008; Sayou et al., 2016; Goslin et al., 2017). It is most obvious on the sides of the inflorescence meristem in floral anlagen and in floral primordia at the earliest stages of development (Weigel et al., 1992). Therefore, our aim in this research is to protect the cauliflower plant from these attacks; a new strain of *Agrobacterium pusense*, JAS1, possesses some compounds that act as plant-growth-promoting bacteria (Kaur et al., 2022). It also makes ammonia, 1-aminocyclopropane-1-carboxylic acid, indole-3-acetic acid, gibberellic acid, and siderophores, which help with fixing nitrogen, making biofilms. It helps in immune boosting of plants, growing root hairs, and shoot growth (Soni et al., 2018; Sagar et al., 2020; Gopalakrishnan et al., 2018). 1-Aminocyclopropanecarboxylic acid is a non-proteinogenic alpha-amino acid composed of a cyclopropane containing both amino and carboxy groups at the 1-position. It is a tautomer of a zwitterion of 1-aminocyclopropanecarboxylic acid (National Center for Biotechnology Information, 2023). Importantly, in our recent study on maize (*Zea mays* L.), we explored the role of 1-amino-cyclopropane-1-carboxylic acid (ACCA) in enhancing plant resistance. We found that ACCA, with a binding energy of −9.98 kcal/mol, effectively strengthens maize against pathogens and drought stress (Debnath et al., 2023a). This finding is pivotal for our current research on cauliflower, as it suggests ACCA’s broader applicability in different plant species.

The importance of enhancing crop resilience, especially for cauliflower, is a key focus in the field of agriculture (Yu et al., 2022). This crop faces numerous challenges, including the threat of pests and environmental stressors. Our study aims to explore the potential of 1-aminocyclopropane-1-carboxylic acid (ACCA) in addressing these issues, building upon the encouraging results from initial computational screenings. While acknowledging the limitations and potential biases of computational models, our research goes beyond identifying promising compounds to understand their effectiveness in real-world agricultural settings. As we embark on this research, our primary goal is to validate the effects of ACCA scientifically and to expand our study to include a wider range of microbial and environmental threats. By doing so, we aim to gain a comprehensive understanding of ACCA’s role in enhancing crop resilience, equipping businesses to better manage both current and future agricultural challenges. This endeavor not only contributes to the scientific community but also has practical implications for sustainable agriculture and food security.

## Materials and methodology

### Target protein and ligand preparation

**Figure 1:**
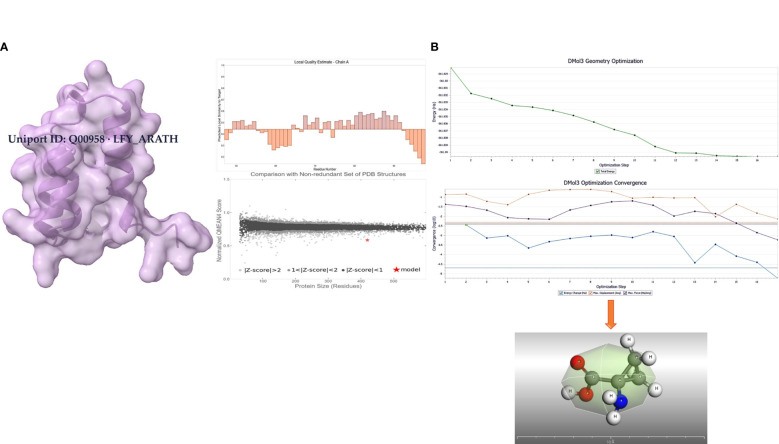
**(A)** Modeled protein with Alphafold2 (Uniport ID: Q00958 LFY_ARATH). **(B)** Geometrically optimized 1-amino-cyclopropane-1-carboxylic acid using DMol3.

The transcription factor protein was retrieved from uniport as FASTA sequence (https://www.uniprot.org/uniprotkb/Q00958/entry); then, it was homologically modeled by Alphafold2 (as depicted in **Figure 1A**). 3D structures in the form of.sdf files were retrieved from PubChem to ascertain what purpose LFY (Uniport ID: Q00958 LFY_ARATH) serves when it binds to the.sdf file for 1-amino-cyclopropane-1-carboxylic acid (ACCA) (Chem I. D: 535) was retrieved from PubChem. The Chimera UCSF team used a 900-step conjugate gradient of energy minimization approach, and a 1,000-step steepest-descent technique was utilized for this optimization. After that, the molecules were converted into.pdb format using a tool called Open Babel. The steepest descent algorithm is then used for a thousand iterations to reduce the molecules’ energy. The AMBER ffSB14 force field is added after the Gasteiger charges have been introduced to set the partial charges. The ligand was further geometrically optimized at DMol3 (as shown in **Figure 1B**).

### Virtual screening of naturally occurring chemicals

In order to establish a connection between ligands and the active site of the protein, we employed BIOVIA Discovery Studio Visualizer version 2022 (Ferreira et al., 2015). This intervention facilitated the achievement of our goal, resulting in the final product exhibiting a significant binding affinity. The software AutoDock Vina was employed to determine the binding site of the protein complex and generate the receptor grid. The ligands were virtually screened using AutoDock Vina 4.2.6. The ligand with the most significant binding energy scores with the transcription macromolecule LFY was chosen. The optimal binding conformation with the highest binding energy was chosen for subsequent redocking and additional investigation for each ligand.

### Molecular re-docking investigations

The receptor grid was constructed using AutoDock MGL version 1.5.6 after virtual screening, with the use of 1-amino-cyclopropane-1-carboxylic acid (ACCA) as the most potent molecule. MGL preserves receptors and ligands in the.pdbqt file format. The spacing between grid points was measured to be 0.57 Å, while the level of exhaustiveness was set at 8. The output files in.pdbqt format were analyzed using PyMol in conjunction with Discovery Studio Visualizer 2021. The co-crystallization of the ligand resulted in the validation and enhancement of ligand binding. Target protein molecules facilitate the binding of 1-amino-cyclopropane-1-carboxylic acid (ACCA). The structure was simplified using the steepest descent approach with a total of 1,000 steps, before applying the AMBER ff4 force field. The molecular docking studies were conducted by researchers utilizing AutoDock 4.2.6 (Toukmaji and Board, 1996; Umar et al., 2022). The formation of the receptor and ligands was facilitated by polar hydrogen bonds, Kollman and Gastieger charges, and electrostatic forces. Following the combination of nonpolar hydrogens, the receptor, and ligand molecules were stored in the.pdbqt file format. Upon the amalgamation of non-polar hydrogen atoms. A grid box was constructed with X=20, Y=15, and Z=26, using a spacing of 0.57Å. The Lamarckian Genetic Algorithm was employed to dock protein–ligand complexes, prioritizing those with the lowest binding free energy (ΔG).

### Molecular dynamics simulation

The molecular dynamics simulation was conducted using the Desmond program, created by Schrodinger LLC (Bowers et al., 2006), to simulate molecular dynamics during a time period of 100 ns. The initial step in initiating the generation of molecular dynamics simulations, encompassing protein and ligand complexes, was doing docking studies. The performance of molecular docking studies in accurately predicting the binding status of ligands is observed to be reliable when conducted inside a static environment. In molecular dynamics (MD) simulations, a commonly employed approach to monitor the temporal evolution of atomic motion involves the integration of the classical equation of motion. Docking provides a fixed representation of the conformation of a molecule’s binding pose within the active site of a protein (Debnath et al., 2023b; Jorgensen et al., 1996). Due to this factor, it is important to give careful thought to this matter. Simulations were employed to examine the physiological condition of ligand binding. The program System Builder was employed in the process of system construction. The transferrable intermolecular interaction potential utilized in each of the three locations was the orthorhombic box model of the solvent. The OPLS 2005-derived force field was employed during the simulation (Xu et al., 2023). The physiological circumstances present during the experiment were simulated by introducing a concentration of 0.15 M by the utilization of NaCl (Debnath et al., 2023a). The simulation was conducted at a temperature of 300 K and a pressure equivalent to atmospheric pressure. Prior to the initiation of the simulation, the models were afforded supplementary autonomy. The evaluation of the model’s consistency involved monitoring the changes in the root mean square deviation (RMSD) of the protein and ligand during the simulation. Trajectories were recorded at regular intervals of 100 ps for further analysis (Mukerjee et al., 2022; Shaw et al., 2010).

## Results

### Virtual screening for most potent naturally occurring compounds

The ligand exhibiting the lowest binding energy score corresponds to the ligand displaying the best binding affinity towards the targeted protein. The ligand’s score, in the presence of 1-amino-cyclopropane-1-carboxylic acid (ACCA), is determined to be −8.74 kcal/mol. The binding cavity was utilized for conducting further reassembly procedures on the molecule exhibiting the highest level of potential. **Table 1** presents the results of a screening conducted on seven ligands to determine their potency in binding to the receptor transcription protein LFY. The molecule depicted in the table exhibits the best efficacy and possesses the greatest potential affinity for the protein under investigation.

**Table 1:** This table showcases a selection of seven compounds along with their respective docking scores measured in kcal/mol.

Ligands	[ΔG] (kcal/mol)
Gibberellic acid	−3.24
Indole-3-acetic acid	−6.24
Citric acid (as siderophore)	−5.47
Hydroxamate	−4.72
**1-Amino-cyclopropane-1-carboxylic acid (ACCA)**	**−8.74**
Dextran	−5.12
Xanthan	−6.27

Notably, the ligand 1-amino-cyclopropane-1-carboxylic acid (ACCA) binds to the core pocket of LFY, exhibiting a binding free energy of −8.74 kcal/mol. This particular entry is highlighted with bold lettering and a red color indication.

### Molecular re-docking

**Figure 2:**
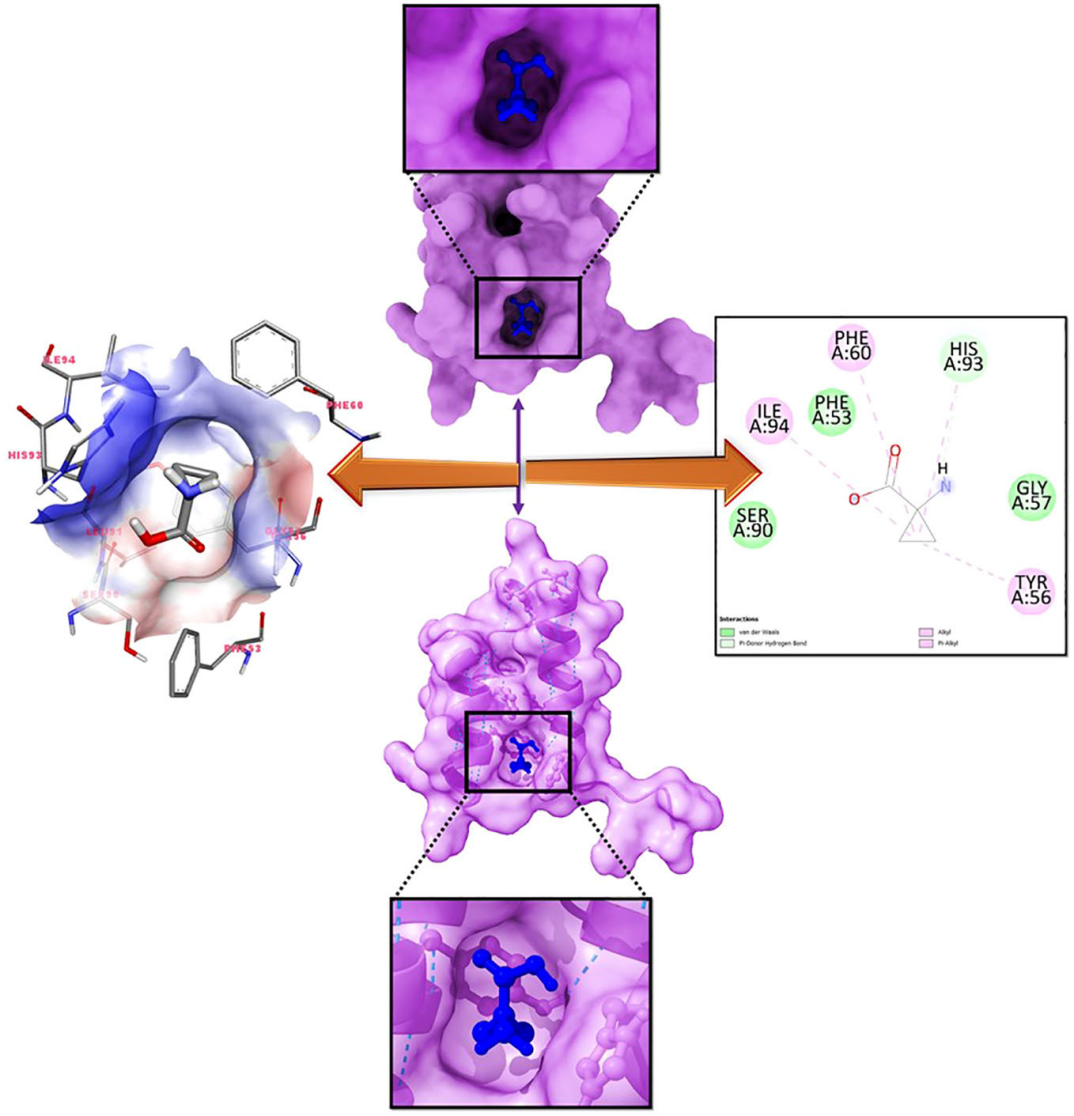
Molecular binding analysis of LFY with 1-amino-cyclopropane-1-carboxylic acid (ACCA) depicted in the 2-D interaction diagram on the right panel.

Molecular docking is a computational technique used to discover the optimal intermolecular arrangement between a macromolecule and a small molecular competitor. In order to ascertain the optimal intermolecular interaction between the target protein and ligand, a molecular docking analysis was conducted. The docking of seven compounds with three-dimensional structures to the target protein was facilitated using the AutoDock Vina wizard and PyRx tools. The binding affinities of the seven ligands are presented in **Table 1**. During re-docking testing, it was shown that 1-amino-cyclopropane-1-carboxylic acid (ACCA) and LFY exhibited a discernible binding pocket, as illustrated in **Figure 2**.

### Molecular dynamics simulation

**Figure 3:**
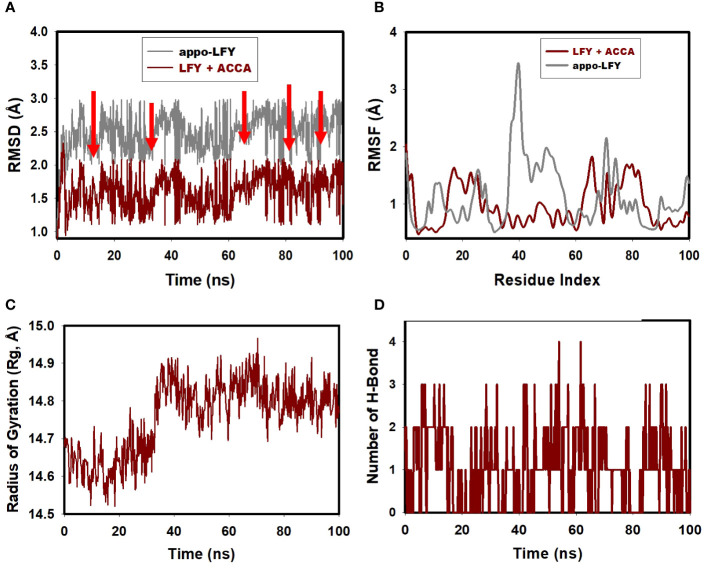
**(A)** LFY+ACCA’s RMSD post 100 ns simulation, illustrated in dark red; **(B)** 100 ns simulation analysis of LFY+ACCA’s RMSF, depicted in dark red; **(C)** gyration radius analysis for LFY+ACCA (shown in dark red) following a 100-ns run; **(D)** hydrogen bond analysis of LFY+ACCA, highlighted in dark red, post 100 ns simulation.

Molecular dynamics (MD) simulations were conducted for a duration of 100 ns. The purpose of this study was to assess the quality and stability of a complex until it reached convergence. Specifically, the root mean square deviation (RMSD) of the Cα-backbone of the transcription protein LFY bound to ACCA was examined. The analysis revealed a deviation of 1.5 Å, as indicated by the dark red colored graph in **Figure 3A**, representing the RMSD.

After 100 ns, the protein exhibits notable fluctuations compared to its reference structure. The average disparity between the protein and the residue positions of the transcription protein LFY bound to ACCA is observed to fluctuate within the range of residues 17–24, as depicted by the dark red colored graph in **Figure 3B**, which represents the root mean square fluctuation (RMSF) graph. The plot displaying the radius of gyration (Rg) of the C-alpha backbone is presented in **Figure 3**, as depicted in the radius of gyration graph. The LFY protein demonstrated a binding affinity to ACCA, as evidenced by a 0.24-Å displacement (indicated by the dark red colored graph) seen during the whole 100 ns simulation, as depicted in the Rg plot of the C-alpha backbone. According to **Figure 3D**, the transcription protein LFY +ACCA exhibits a single instance of hydrogen bonding over a 100-ns simulation. Consequently, this led to the successful maintenance of stability inside the simulation.

### MM-GBSA calculations

The binding free energy and other contributing energy components, such as MM-GBSA, were computed for each LFY + ACCA complex by utilizing the molecular dynamics (MD) simulation trajectory. The findings shown in **Table 2** indicate that ΔGbindCoulomb, ΔGbindvdW, and ΔGbindLipo made the most significant contributions to the stability of the simulated complexes. Conversely, ΔGbindCovalent and ΔGbindSolvGB were found to contribute to the instability of these complexes.

**Table 2:** Determination of binding free energy components for the LFY and ACCA combination using MM-GBSA method.

Various Energies (kcal/mol)	ACCA
**ΔG_bind_**	−45.126 ± 3.27
**ΔG_bind_Lipo**	−23.25 ± 1.12
**ΔG_bind_vdW**	−10.12 ± 1.3
**ΔG_bind_Coulomb**	23.67 ± 0.27
**ΔG_bind_H_bond_**	−30.78 ± 1.72
**ΔG_bind_SolvGB**	0.45 ± 1.99
**ΔG_bind_Covalent**	3.14 ± 0.23

**Figure 4:**
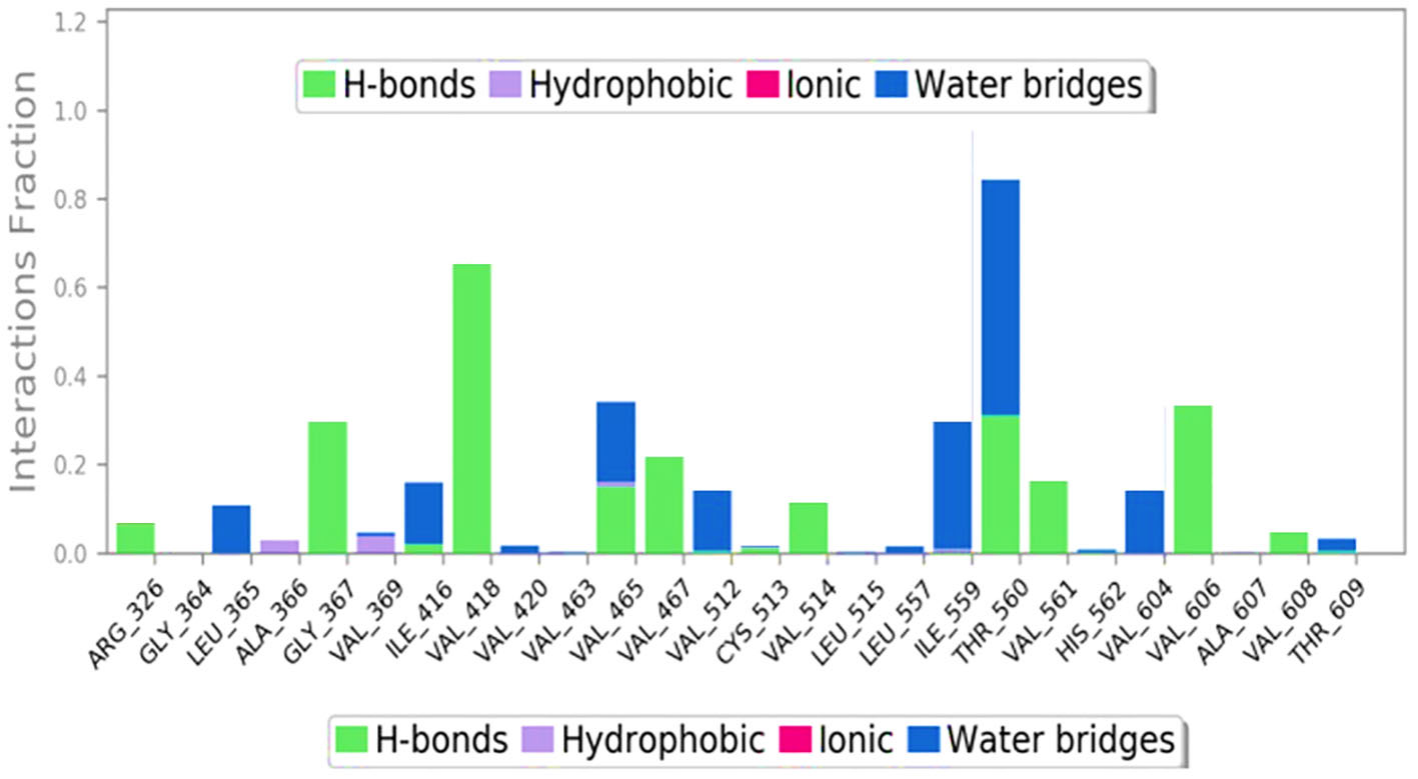
Various types of bonds formed during 100 ns run.

It is evident that ACCA has established robust hydrogen bonds upon interacting with the residues previously anticipated to be bound (as depicted in **Figure 4**). Additionally, it has been demonstrated that a multitude of non-covalent interactions, including as hydrophobic interactions, ionic interactions, hydrogen bonding, and water bridges, are present.

**Figure 5:**
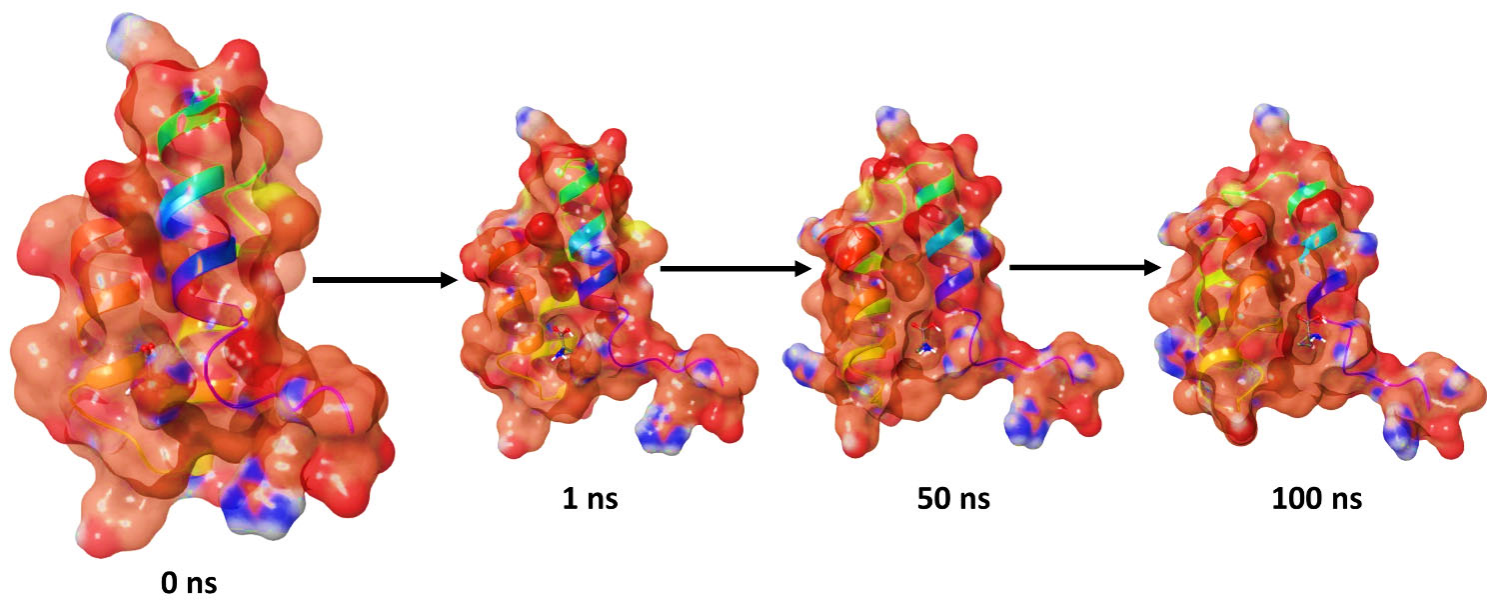
Stepwise interaction analysis after 100 ns run.

From **Figure 5**, its can be clearly stated that over the course of 100 ns run, the ligand binds compactly with the LFY protein.

## Discussion

Cauliflower, a crop highly sensitive to environmental conditions, often suffers from water stress, especially in hot weather conditions, leading to reduced yield and quality. Additionally, nitrogen scarcity can severely impact its growth. In such contexts, enhancing drought tolerance and nutrient uptake efficiency becomes crucial. Our computational analysis identified ACCA as a promising compound that interacts with the transcription factor LEAFY (LFY), known for its critical role in the early stages of flower development (Weigel et al., 1992; Hamès et al., 2008; Sayou et al., 2016; Goslin et al., 2017). By modulating LFY activity, ACCA may contribute to the plant’s resilience against environmental stresses.

LFY encodes a plant-specific transcription factor predominantly active in the inflorescence meristem and floral primordia at the earliest development stages (Weigel et al., 1992). This suggests that influencing LFY activity through ACCA could have significant implications for flower development under stress conditions. The computational studies indicated a strong interaction between LFY and ACCA, suggesting potential benefits in enhancing drought tolerance and possibly improving nutrient assimilation. Moreover, previous research on a new strain of *Agrobacterium pusense*, JAS1, which produces chemical compounds, further reinforces the potential of ACCA (Kaur et al., 2022). The integration of computational technologies into plant science has been instrumental in advancing our understanding of complex biological processes. The computational approach in our research offers significant benefits, including the rapid and cost-effective assessment of various compounds. This method has led us to identify ACCA as a promising agent, particularly in its affinity for LFY, evidenced by a substantial binding free energy of −8.74 kcal/mol (Yu et al., 2022). This binding energy is a critical indicator of the strength and stability of the ligand–protein interaction, essential for the modulation of biological processes.

Our in-depth computational examination of the LFY-ACCA complex revealed important insights. We focused on understanding the roles of various molecular interactions, such as lipophilic, van der Waals, and hydrogen bonds, in maintaining the stability of this complex (Kaur et al., 2022). Moreover, molecular dynamics simulations provided predictions on the temporal behavior of the LFY-ACCA complex, consistently indicating its stability, which is pivotal for the prolonged and effective modulation of LFY function.

However, while computational models are invaluable, they do not replace the need for empirical validation. Although these models can offer extensive theoretical insights, they may not capture all the nuances of biological systems in natural environments. The encouraging results with ACCA underscore the necessity for experimental studies to validate its effectiveness in enhancing drought tolerance in cauliflower.

The potential application of our findings is significant, particularly in the context of agricultural sustainability. Utilizing natural compounds like ACCA to improve drought tolerance in crops presents an environmentally friendly approach, in line with global conservation efforts (Ferreira et al., 2015). Improving drought resistance in crops such as cauliflower can lead to increased yield and productivity, addressing critical issues like food security and providing economic advantages to farmers (Meng et al., 2011).

Thus, our study emphasizes the potential of ACCA in interacting with LFY to enhance drought tolerance in *Brassica oleracea* var. *botrytis*. It demonstrates the power of computational tools in plant biology research and sets the stage for necessary empirical studies. Moving forward, it is crucial to undertake experimental validations to confirm the computational predictions and explore the wider implications of ACCA in improving drought resilience in various crops, marking a significant step forward in integrated plant science research.

## Conclusion

In our study, we have delved into the enhancement of cauliflower’s resilience to environmental stressors, a vital consideration given its vulnerability to various challenges. Through the use of advanced computational methods, we embarked on virtual screening to pinpoint robust natural compounds capable of fortifying cauliflower’s defenses. Among these, 1-aminocyclopropane-1-carboxylic acid (ACCA) stood out as an exceptionally promising candidate, notable for its unique molecular structure and its interaction with the LEAFY (LFY) protein, integral to the plant’s developmental processes.

ACCA, characterized by its non-proteinogenic alpha-amino acid structure that incorporates a cyclopropane ring with functional groups at the 1-position, exhibits a strong affinity towards the LFY protein. This interaction is crucial considering LFY’s significant role in the growth dynamics of cauliflower, particularly in its flowering mechanisms. The binding of ACCA to LFY not only suggests an enhancement of the plant’s natural defense mechanisms against pathogens but also underscores ACCA’s role in bolstering the plant’s resilience to environmental stresses, especially drought.

Moreover, our research highlights ACCA’s potential in ameliorating drought tolerance in cauliflower. This is particularly pertinent in light of the cultivation challenges of cauliflower, such as water scarcity and nutrient deficiency. The molecular properties of ACCA, including its lipophilic tendencies and hydrogen bonding capabilities, contribute significantly to its effectiveness in boosting the plant’s overall stress resilience. Thus, our findings point towards ACCA as an environmentally friendly and efficacious tool in agricultural science, paving the way for more sustainable farming practices, improved crop yields, and heightened resilience of cauliflower against both biotic and abiotic stressors.

While our results are encouraging, they represent the inception of an extensive exploration into the wider implications of ACCA’s application in agriculture. The forthcoming phases of our research will focus on the empirical validation of these computational predictions, with the ultimate aim of translating this theoretical knowledge into tangible, practical agricultural applications. This endeavor not only contributes to the scientific community but also holds significant promise for sustainable agriculture and food security.

## Data availability statement

The datasets presented in this study can be found in online repositories. The names of the repository/repositories and accession number(s) can be found in the article/supplementary material.

## Author contributions

VS: Data curation, Investigation, Project administration, Supervision, Validation, Visualization, Writing – original draft, Writing – review & editing. SD: Data curation, Conceptualization, Investigation, Software, Validation, Visualization, Writing – review & editing. AS: Investigation, Supervision, Validation, Visualization, Writing – review & editing. AB: Conceptualization, Data curation, Validation, Visualization, Writing – review & editing. RE: Validation, Visualization, Writing – review & editing. MV: Investigation, Project administration, Validation, Visualization, Writing – review & editing. CK: Data curation, Investigation, Project administration, Visualization, Writing – review & editing. LW: Project administration, Supervision, Validation, Visualization, Writing – review & editing. VK: Investigation, Methodology, Validation, Visualization, Writing – review & editing. AE: Data curation, Formal analysis, Software, Visualization, Writing – review & editing.
